# The Association Between Maternal Diet and the Human Milk Microbiome: A Review of Evidence and Methodological Challenges

**DOI:** 10.3390/microorganisms13102347

**Published:** 2025-10-14

**Authors:** Azhar S. Sindi

**Affiliations:** Department of Clinical Laboratory Sciences, College of Applied Medical Sciences, Umm Al-Qura University, Makkah 24381, Saudi Arabia; asmsindi@uqu.edu.sa; Tel.: +966-565611326

**Keywords:** maternal diet, maternal nutrition, human milk, microbiota, microbiome

## Abstract

The human milk (HM) microbiome plays an important role in shaping the infant gut microbiota, with potential implications for immune development and both short- and long-term health. Among the maternal and infant factors influencing HM microbial composition, maternal diet represents a modifiable determinant. However, evidence regarding the impact of diet on the HM microbiota remains limited, and the methodological quality of available studies is variable. This review synthesises findings from 15 observational and interventional studies, critically evaluating dietary assessment approaches, milk collection protocols, microbiome analysis methods, and control of confounding factors. Current evidence suggests that maternal intake of macronutrients, micronutrients, and bioactive compounds may influence HM bacterial composition and functional potential, though results are inconsistent. Key limitations across studies include small sample sizes, short intervention periods, lack of appropriate control groups, variable aseptic sampling methods, inadequate contamination controls, and insufficient adjustment for confounders. To advance the field, we recommend larger, multicentre randomised controlled trials with longer intervention durations, incorporation of dietary biomarkers, standardised HM collection and processing protocols, and advanced multi-omics approaches. Strengthening methodological rigour is essential to generate robust evidence that can guide dietary interventions aimed at optimising the HM microbiota and improving infant health outcomes.

## 1. Introduction

The colonisation of the infant gut during early life is a dynamic process shaped by both environmental and host-related factors. Key determinants of the infant gut microbiome include mode of delivery [1,2], infant feeding method [3,4,5,6], and exposure to antibiotics [1]. This early microbial colonisation undergoes rapid shifts, marked by increasing bacterial diversity. In breastfed infants gut, *Bifidobacterium* spp. are the dominant, whereas formula-fed infants show a more diverse microbial profile [7]. The infant gut microbiota can protect infants from enteric pathogens and contribute to the development of the immune system [8,9]. Results from previous studies suggest that perturbations to the early-life gut microbiome are associated with later-life disease such as allergic disease [10,11,12,13,14,15], obesity [16], and type 1 diabetes [17,18].

**Figure 1:**
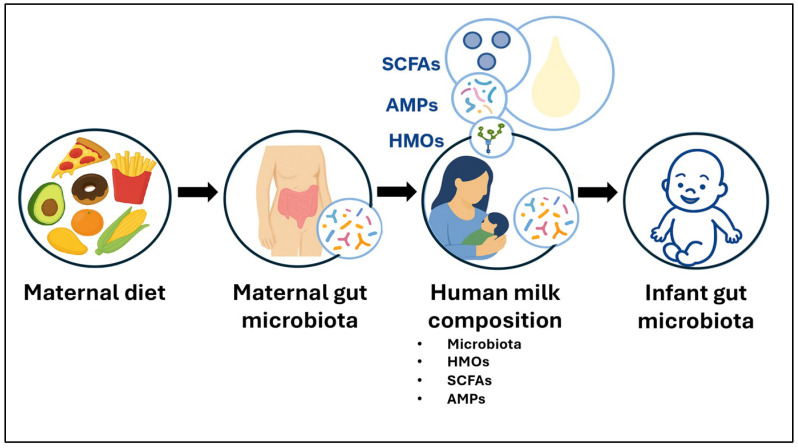
Conceptual pathway linking maternal diet to the infant gut microbiota.

The human milk (HM) microbiome is an important factor that contribute to the infant gut colonisation, together with other several factors such as solid food introduction, mode of delivery, geographical location, household siblings and furry pets [2,6,19]. Several studies have detected microbial species, such as *Bifidobacterium* spp. and bacteriophages, that are shared to both HM and infant gut paired samples [20,21,22,23,24,25]. The application of high-resolution profiling methods, such as whole genome shotgun sequencing and single nucleotide variant analysis has provided robust evidence of strain-level sharing between HM and the infant gut microbiome. Nevertheless, this does not definitively indicate vertical transmission, as both sources can simply be exposed to the same environmental source of microbes. In addition to the beneficial effect of the HM mediated by the transfer of microbial components to the breastfed infants gut [20,21,22,23,24,26], other bioactive factors are also delivered, including human milk oligosaccharides (HMOs) [27,28], antimicrobial peptides (AMPs) [29], and bacterial metabolites (Figure 1) [27,28,29,30,31]. Consequently, the gut microbiome of breastfed infants differs from that of formula-fed infants [6,32], highlighting the important role of HM in shaping early gut microbial colonisation.

Maternal dietary patterns shape the maternal gut microbiota, which contributes to the human milk composition, including its microbiota and bioactive factors such as HMOs, short-chain fatty acids (SCFAs), and antimicrobial peptides (AMPs). These milk components can seed and modulate the infant gut microbiota.

While several maternal factors such as mode of delivery [33,34,35,36,37,38,39,40,41], lactation stage [33,34,42,43,44,45,46], geographical location [36,38,41,42,47], breastfeeding practices [40,45,48,49,50] and infant factors, including gestational age at delivery [34], infant sex [40,51], and infant allergy [52,53] have also been associated with the HM microbiota composition, maternal diet is of particular interest because it represents a modifiable exposure during the perinatal and postpartum period. Diet may influence the HM microbiota by altering the maternal gut microbial composition or through changes in HM components such as HMOs, which in turn may modulate the HM microbiota (Figure 1). This underscores the potential of maternal diet as a promising target for interventions aimed at promoting optimal infant health through modulation of the HM microbiota.

Despite growing interest in this field, evidence on the impact of maternal diet on the HM microbiome remains limited and heterogeneous. A recent scoping review by Taylor et al. [54] systematically identified 19 eligible studies addressing the association between maternal diet and either the HM or infant gut microbiome. Of these, only 10 studies assessed the HM microbiome directly, and only two evaluated both HM and infant gut microbiota concurrently. Findings across studies varied widely in terms of dietary exposures, microbial outcomes, and methodological rigor, with many reporting correlations between specific dietary components such as polyunsaturated fatty acids, fibre, and micronutrients and the relative abundance of bacterial taxa in HM [54]. To date, no review has critically appraised the robustness and reproducibility of the existing evidence linking maternal diet to the HM microbiome. Given the increasing number of observational and interventional studies in this area, it is essential to assess the methodological quality, biological plausibility, and consistency of reported associations. This narrative review therefore critically evaluates current evidence to clarify whether maternal diet may shape the HM microbiome, identifies key limitations in previous studies design and methods for both microbiome analysis and maternal dietary assessment, and outlines future directions to strengthen the evidence base.

## 2. Method

Relevant studies were identified through searches in PubMed, ProQuest, and Google Scholar up to August 2025, using combinations of terms related to maternal diet, human milk, and microbiota. Additional studies were extracted from the reference lists of relevant articles. The focus was on studies directly examining associations between maternal dietary intake and the HM microbiome. The search covers all literature published up to August 2025.

## 3. The Human Milk Microbiota Composition and Its Determinants

HM is a complex biological fluid that contains essential nutrients, immune-modulating factors, and diverse microbial communities [55,56,57]. It contributes to the initial colonisation of both the infant oral [58] and gut microbiota [20,22,23,25,59]. Several bacterial species have been identified as common to HM and the infant gut, including *Bifidobacterium breve*, *B. adolescentis*, *B. dentium*, *B. infantis*, *B. longum*, *B. bifidum*, *B. angulatum*, *Staphylococcus epidermidis*, and *Veillonella parvula*. Similarly, species such as *S. epidermidis*, *S. auricularis*, *Streptococcus parasanguinis/gordonii*, *S. mitis/oralis*, and *S. salivarius* are frequently shared between HM and the infant oral cavity [20,23,24,25]. Bacterial species in HM are most commonly characterised using 16S rRNA gene sequencing (targeting hypervariable regions such as V1–V2, V3–V4, or V4) or less frequently, by shotgun metagenomic sequencing [20,21,26]. HM has also been reported to be dominated by *Malassezia* and lower levels of other fungal species such as *Davidiella*, *Candida*, and *Saccharomyces* [60,61]. Fungal sequences from the phyla Basidiomycota and Ascomycota were detected in HM from both healthy women and those with mastitis [26]. These fungal profiles were identified using a range of methods, including microscopy, cultivation, quantitative PCR, ITS rRNA, 18S rRNA, 28S rRNA gene sequencing, and shotgun metagenomics [26,60,61]. However, other microorganisms such as viruses and archaea have also been reported in HM [23,26,62,63].

The composition of the HM microbiota shows significant inter-individual variability [64]. Nevertheless, *Staphylococcus* spp. and *Streptococcus* spp. are consistently reported as dominant genera across previous studies [21,26,65,66,67,68,69], while the abundance and prevalence of other bacterial taxa vary widely among individuals and populations [47]. A range of maternal and infant-related factors have been associated with the HM microbial composition. Maternal factors include mode of delivery [33,34,35,36,37,38,39,40,41], lactation stage [33,34,42,43,44,45,46], maternal body mass index (BMI), [33,36,41,45,51,70,71,72,73], geographical location [36,38,41,42,47], breastfeeding practices [40,45,48,49,50], parity [40,45], and allergy [40,74]. Infant related factors include gestational age at delivery [34], infant sex [40,51], and infant allergy [52,53]. Given the important role of HM microbiota in the infant gut colonisation and immune development, a comprehensive understanding of factors influencing its composition is essential for guiding strategies aimed at promoting infant health through targeted maternal and infant interventions.

## 4. Origin of Human Milk Bacteria

The HM microbiota is increasingly recognised as originating from multiple sources, including both internal and external sources. Two major mechanisms have been proposed to explain the presence of bacteria in HM, the retrograde transfer of microbes from the infant’s oral cavity and maternal skin during breastfeeding, and entero-mammary pathway [75,76,77,78]. Retrograde milk flow from the infant’s mouth into the breast has been observed during breastfeeding and is hypothesised to occur during milk expression using breast pumps [79]. This bidirectional movement may facilitate the transfer of infant oral, maternal skin, and/or pump-associated bacteria into the mammary ducts. The mode of feeding, whether through direct breastfeeding or the use of expressed milk, has been consistently associated with the composition of the HM microbiota [40,45,48,49,50]. Furthermore, the increased risk of developing lactational mastitis among women who use breast pump supports the hypothesis that mechanical expression may introduce or promote colonisation by external bacteria [80,81]. However, a recent study comparing aseptically and non-aseptically collected HM samples found that typical skin and oral bacteria such as *Staphylococcus* spp., *Streptococcus* spp., and *Rothia* spp. were present even under sterile sampling conditions, supporting their role as true members of the HM microbiota rather than external contaminants [82].

Among proposed internal sources, the most robust evidence supports the maternal gut as a contributor via the entero-mammary pathway. The presence of obligate anaerobes such as *Bacteroides* spp. and *Bifidobacterium* spp. in HM suggests their transfer from the gut [25,69,78], along with studies showing shared bacterial strains between maternal gut and HM [22,23,24] provide strong support for this route. However, a shared environmental source might exist rather than vertical transmission. Additionally, most studies investigating probiotic supplementation during pregnancy and lactation report successful detection of the supplemented strains in HM [83,84,85,86,87,88,89], though colonisation levels vary between individuals. This variation may be influenced by several factors, including maternal gut microbiota composition, immune status, and genetic differences, the specific probiotic strain used, dosage, duration of supplementation, timing of sampling, and physiological differences in gastrointestinal transit or permeability during pregnancy and lactation [90]. Further research is needed to clarify host and microbial factors associated with the probiotic colonisation of HM.

Bacterial translocation is a physiological process that has been shown to increase during pregnancy and lactation where bacteria from maternal gut are translocated to mesenteric lymph nodes and mammary tissue. Evidence from animal models suggests that intestinal bacteria may be transported to the lactating mammary gland via immune cells such as dendritic cells [91,92,93]. These immune cells can sample bacteria from the maternal gut by crossing intestinal epithelial tight junctions while maintaining barrier integrity through the expression of tight junction proteins [94]. In humans, the temporary opening of mammary epithelial tight junctions during pregnancy and the early postpartum period may facilitate bacterial entry into mammary tissue [95]. However, the exact mechanism by which bacteria cross from the lymphatic system into the mammary gland remains unclear.

In addition to direct bacterial translocation, maternal gut bacteria may influence the HM microbial composition through the production of metabolites such as SCFAs. SCFAs are primarily produced by the gut microbiota during the fermentation of dietary fibres mainly in the colon, absorbed into the maternal bloodstream, and may subsequently reach the mammary gland [96]. These metabolites may also be synthesised locally by mammary-resident bacteria. Low SCFAs levels in HM have been associated with maternal atopy [97] and fat formation and fat cell metabolism in infants [98], suggesting their potential role in infant development and health. Moreover, SCFAs concentrations in HM have been associated with the composition of both the HM microbiota and the infant gut microbiota [99], highlighting their significance in shaping early-life microbial colonisation and infant health outcomes. However, studies directly investigating SCFAs in HM and their effects on infant health and development remain limited.

SCFA production in the maternal gut and their subsequent absorption into the bloodstream are modulated by maternal diet, particularly dietary fibre intake [100,101,102,103,104,105]. These diet-derived SCF may in turn affect SCFAs levels in HM, further supporting a functional connection between the maternal gut microbiota and HM composition. While existing evidence supports the maternal gut as a source of both bacteria and their metabolites in HM, the specificity, regulation, and overall contribution of this pathway remain incompletely understood and require further investigation.

## 5. Studies Investigating the Association Between Maternal Diet and the Human Milk Microbiota

To date, fifteen studies have investigated the association between maternal diet and the HM bacterial composition [40,50,51,73,106,107,108,109,110,111,112,113,114,115,116]. The majority of these studies reported significant associations [50,51,73,106,107,108,109,110,111,112,113], whereas only four found no clear relationship [40,114,115,116]. Of the fifteen studies, four were interventional [109,111,114,116], while the rest were observational studies [40,50,51,73,106,107,108,110,112,113,115]. In a longitudinal study, Williams et al. analysed HM samples collected at nine different time points postpartum (days 2, 5, and 10, and months 1 through 6) from a cohort of 21 healthy breastfeeding women [51]. Maternal dietary intake was captured at each sampling point using a 24-h dietary recall. The study identified several associations between maternal diet and the composition of the HM microbiota (Table S1). However, one notable limitation, as highlighted by the authors, was the use of averaged values for both dietary intake and microbial profiles across all time points (Table S2). This approach may mask temporal variations, particularly since the sampling period included the transition from colostrum to transitional and mature milk stages, which are known to have distinct bacterial composition [33,34,117]. Additionally, both dietary habits and milk microbiota are expected to change over the first six months postpartum, further complicating interpretation of mean-based analyses.

In another longitudinal study involving 22 healthy lactating women from Israel. Babakobi et al. collected HM samples at one week, one month, and three months postpartum [107]. Maternal dietary intake was assessed retrospectively at three months postpartum using a validated food frequency questionnaire (FFQ) that covered both pregnancy and lactation periods. To validate the FFQs, 24-h dietary recalls were also completed one day prior to each sampling. The study identified a negative association between *Streptococcus* spp. abundance in HM and maternal intake of polyunsaturated fat, monounsaturated fat, and folic acid at one month postpartum, while a positive association was observed between vitamin B12 intake and *Streptococcus* spp. abundance at three months postpartum. One limitation of this study is that maternal dietary intake during pregnancy and lactation was combined in the analysis. This limits the ability to identify their individual contributions to the HM bacterial composition. Another limitation is that all mothers declared their intention to practice exclusive breastfeeding until at least three months of age; however, the study did not report whether this was actually achieved, which may confound the observed associations, as breastfeeding exclusivity can influence the HM microbiota composition and potentially bias the results (Table S2) [40,45,49,50].

Nine cross-sectional studies have explored the relationship between maternal diet and the HM microbiota; however, these investigations relied on HM samples collected at only one time point [40,50,73,106,108,110,112,113,115]. For example, Moossavi et al. examined HM samples collected from a large, population-based cohort of 393 healthy lactating women enrolled in the Canadian CHILD study (Table S1) [40]. HM was collected at 3–4 months postpartum, and microbiota composition was assessed using 16S rRNA gene sequencing. Maternal dietary intake was evaluated using an FFQ, although the timing of dietary assessment relative to milk sampling was not reported. Despite the extensive dataset and use of rigorous microbiome profiling methods, maternal diet was not directly associated with HM microbiota composition. Two main limitations of this study include the lack of reporting on dietary assessment timing and the use of non-aseptic milk collection methods, which may increase the risk of skin and other environmental contaminants.

Padilha et al. examined the HM microbiota in 94 Brazilian lactating women at day 30 (±4) postpartum (Table S1) [106]. Maternal diet during lactation was captured using two 24-h dietary recalls at days 7 (±3) and 30 (±4) postpartum, while dietary intake during pregnancy was assessed via an FFQ administered at day 30 (±4) postpartum. Multiple associations were observed between nutrient intake and specific HM bacterial taxa. Notably, vitamin C intake during pregnancy was positively associated with a *Staphylococcus*-driven cluster. During lactation, polyunsaturated fat and linoleic acid consumption were associated with increased *Bifidobacterium* spp. in the HM. *Pseudomonas* spp. were enriched in the HM of mothers with lower sugar intake and higher intake of vitamin B9, whereas *Enterococcus* spp. abundance decreased with increased B vitamins (B1, B2, and B9) intake during lactation. This study is limited by variation in infant feeding practices, as 83% of the infants were exclusively breastfed, while 17% received both HM and formula, which may have confounded the results.

Cortes-Macías et al. characterised HM microbiota from 120 healthy Spanish women at 11 (±4) days postpartum (Table S1) [73]. Maternal diet during pregnancy was evaluated using an FFQ administered at the same time of milk sampling. Participants were clustered into two dietary patterns: Cluster I characterised by higher intake of fibre, plant protein, and carbohydrates, and Cluster II by greater consumption of animal protein and lipids. Several associations were detected between dietary components and HM microbiota. For instance, *Bifidobacterium* spp. abundance was positively linked to higher polyphenol and carbohydrate intake and negatively associated with total lipid intake. The study also reported that mode of delivery and intrapartum antibiotic exposure significantly influenced HM bacterial profiles in a diet-dependent manner. Significant differences in the HM microbiota inferred function were also detected depending on the maternal diet.

LeMay-Nedjelski et al. analysed HM samples from 93 Canadian women with varying metabolic conditions (normoglycemia, gestational diabetes mellitus, and impaired glucose tolerance) at three months postpartum [50]. Dietary intake was assessed using an FFQ. The analysis revealed associations between maternal fat and fibre intake and HM bacterial diversity and composition. For example, fibre intake from grains correlated with increased alpha and beta diversity, as well as decreased *Fusobacteria* spp. and elevated *Acinetobacter* spp. in the HM. Total fibre was linked to lower *Streptococcus* spp. abundance, while trans fat intake was positively associated with *Staphylococcus* spp. and *Gemella* spp. in the HM. Interestingly, while monounsaturated fat was associated with increased *Gemella* spp. and *Acinetobacter* spp., polyunsaturated fat showed an inverse relationship with *Acinetobacter* spp. in the HM. Total fibre consumption was associated with variations in the β-diversity of predicted bacterial functions of the HM. This study is limited by the absence of subgroup analyses for metabolic groups and the inclusion of women with glucose intolerance conditions known to influence the gut bacterial composition [118,119], thereby limiting the generalisability of the conclusions.

Shenker et al. evaluated associations between maternal dietary intake and HM bacterial composition in 62 healthy UK lactating women using a cross-sectional design (Table S1) [115]. Self-reported intake of food groups and supplements such as vitamin D and calcium was collected before the time of HM sampling, which occurred between 3 and 48 months postpartum. 16S rRNA gene sequencing was used to characterise the HM microbiota. However, no significant associations were identified between dietary variables and HM taxonomic profiles. Major limitation of this study is the lack of reporting on key variables, such as exclusive breastfeeding status and gestational age at delivery, which may confound the results (Table S2) [34,40,45,48,49,50,120]. Additionally, the broad and variable timing of dietary assessment and HM sample collection ranging from 3 to 48 months postpartum adds heterogeneity that may affect the interpretation of the results.

Marsh et al. explored differences in HM microbiota among 72 American healthy lactating women based on dietary pattern using a cross-sectional design [108]. Dietary intake was assessed using an FFQ, and HM samples were collected at ≥2 weeks postpartum. HM bacterial profiles were characterised using 16S rRNA gene sequencing. The authors compared HM microbiota profiles among omnivorous, vegetarian, and vegan participants. An omnivore diet was associated with increased abundance of taxa including, *Vermiphilaceae*, *Dietzia*, *Mycobacterium*, *Rothia*, *Prevotellaceae* NK3B31, and *Bilophila*. Conversely, vegan diet was associated with increased *Muribaculum*, *Halobacillus*, *Clostridium spiroforme*, and *Cloacibacterium*. These findings suggest dietary pattern may influence the HM bacterial composition; however, the timing of dietary intake assessment relative to sample collection was not reported, and exclusive breastfeeding status was not specified both of which may have confound the interpretation of results [40,45,48,49,50,120].

Londoño-Sierra et al. examined the associations between maternal dietary intake and HM microbiota among 30 healthy Colombian women using a cross-sectional design (Table S1) [110]. Maternal diet was assessed using an FFQ during the last two trimesters of pregnancy and through two non-consecutive 24-h dietary recalls during the first trimester of lactation. HM samples were collected during the first trimester of lactation and bacterial profiles were identified using 16S rRNA gene sequencing. Several associations were identified. For example, intake of simple carbohydrates was correlated with increased abundance of *Enterobacter* and decreased levels of *Bifidobacterium*. Higher intake of total and saturated fat, as well as monounsaturated fat, was associated with an increase in *Eubacterium* and a reduction in *Bifidobacterium*. Folic acid and B-complex vitamins (B1, B2, B3) were positively associated with *Akkermansia* and *Gemella*, respectively. Multiple additional relationships were reported for other taxa, highlighting the complex interplay between maternal dietary patterns and the HM microbiota composition. However, the specific timing of maternal dietary assessment during gestation and lactation was not reported. Another limitation of this study is that the exact timing of HM sample collection within the first trimester of lactation was not specified, making it unclear whether samples represented colostrum, transitional, or mature milk each of which may differ in bacterial profiles [33,34,42,43,44,45,46].

Ajeeb et al. examined the relationship between maternal dietary intake and HM microbiota among 64 healthy lactating women in Guatemala using a cross-sectional design [112]. Dietary data were collected through two non-consecutive 24-h dietary recalls during early (6–46 days) and late lactation (109–184 days). HM samples were collected on the same day as the second 24-h recall during the late lactation period. The HM microbiota was characterised via 16S rRNA gene sequencing. The authors reported multiple associations between specific dietary factors and microbial taxa. Notably, higher intake of pantothenic acid, choline, saturated fat, cobalamin, riboflavin, cholesterol, and vitamin D were positively associated with *S. salivarius*, *Streptococcus*_MS_12, *Corynebacterium*_1, *Kocuria palustris*, and *Brevundimonas*_MS_1. A major limitation of this study is that while HM samples were collected during the late lactation period (109–184 days postpartum), the study did not account for infant feeding practices (exclusive versus non-exclusive breastfeeding), which have been shown in another study using the same cohort to significantly influence the HM microbiota at the species level (Table S2) [120]. Ajeeb et al. included mothers who exclusively or predominantly breastfed their infants, with predominant breastfeeding defined as breastfeeding accompanied by the use of agüitas (a ritual fluid) for six months, whereas López Leyva et al. (2022) defined non-exclusive breastfeeding as feeding the infant herbal teas (agüitas) and/or complementary foods while continuing to breastfeed [120]. Infant intake of agüitas might be particularly problematic, as López Leyva et al. demonstrated that such non-exclusive feeding practices were associated with distinct alterations in the HM microbiota. Specifically, non-exclusive feeding practices were associated with reduced abundance of commensal and oral-associated bacteria such as *Lactobacillus gasseri*, *Granulicatella elegans*, *S. mitis*, and *S. parasanguinis* and increased presence of environmentally derived taxa [120]. Given that the present study did not control for or stratify by breastfeeding exclusivity, the observed associations between maternal dietary intake and HM microbiota might be confounded by unmeasured variation in feeding practices.

Bzikowska-Jura et al. conducted a pilot cross-sectional study in Poland to explore associations between maternal dietary intake and HM microbiota composition. They recruited 15 healthy lactating women and collected HM samples at 4–6 weeks postpartum. Maternal dietary intake was evaluated using both a semi-structured FFQ to assess the intake during the last two months of pregnancy and one month of lactation, and a 3-day dietary record to measure current intake levels. 16S rRNA gene sequencing was used to characterise HM bacterial profiles. Numerous correlations were observed between maternal intake and HM bacterial taxa. For example, starch, vitamin A, beta-carotene, monounsaturated fatty acids, animal protein, and total carbohydrate intake were positively associated with an increased abundance of *Firmicutes*. Similarly, intake of retinol was positively associated with Bacteroidota. Several other associations were also identified. However, as a pilot study with a small sample size (*n* = 15), the findings should be interpreted with caution due to limited statistical power and reduced generalisability. Additionally, the study combined retrospective and prospective dietary assessment methods to measure maternal dietary intake over a broad period, including both pregnancy and lactation, which may introduce recall bias and may not accurately reflect dietary intake during these distinct physiological stages.

In addition to observational studies, a limited number of interventional studies have examined the impact of maternal dietary intervention on the HM microbiota, offering insights into potential causal relationships. Bisanz et al. conducted an open-label, pilot dietary intervention study in Tanzania to explore the effect of probiotic yogurt intake on the HM microbiota composition (Table S1) [114]. The study included 15 healthy lactating women with term infants, of whom six consumed 250 g of Moringa-supplemented probiotic yogurt daily (6 days/week) during the last two trimesters of pregnancy and for one month postpartum, while the remaining nine served as controls. HM samples were collected between one week and one month postpartum. Maternal dietary intake was assessed using a 48-h recall at gestational weeks 21 ± 4 and 32 ± 2, and one week to one month postpartum. Microbial profiling was performed using 16S rRNA gene sequencing. The probiotic yogurt delivered approximately 10^10^ CFU of *Lactobacillus rhamnosus* GR-1 per serving along with protein, calcium, vitamin A, B2, and iron. However, the intervention did not result in significant differences in HM microbiota composition or diversity between the intervention and control groups. Despite its strengths, the study is limited by its small sample size and underpowered design, which may have hindered the ability to detect meaningful microbial differences (Table S2). Additionally, participants in the probiotic group consumed yogurt for an average of 88 ± 31 days, indicating considerable variability in intervention duration. Another limitation of this study is the variability in the timing of HM sample collection, which ranged from 1 week to 1 month postpartum. As a result, the samples encompass both transitional and mature milk, each of which may differ in their bacterial composition, potentially confounding comparisons and interpretation of the data [33,34,42,43,44,45,46].

Seferovic et al. conducted two randomised single-blinded cross-over dietary interventions in 14 healthy American lactating women to investigate the short-term effects of maternal diet on the HM microbiota. In the Glu/Gal cohort (*n* = 7), participants consumed isocaloric drinks containing glucose or galactose for 30–57 h. The Carb/Fat cohort (*n* = 7) followed either a high-carbohydrate (60%) or high-fat (55%) diet for 8 days. HM samples were collected at the end of each intervention and analysed by shotgun metagenomic sequencing and 16S rRNA gene sequencing. While taxonomic composition remained largely unchanged, both interventions significantly altered the metagenomic functional profile of HM bacteria, suggesting that maternal diet can modulate microbial function independently of taxonomic shifts. However, a few limitations should be considered. First, functional pathways were inferred from metagenomic data without direct assessment of microbial gene expression or protein activity, limiting conclusions regarding the actual metabolic function of HM bacterial communities. Additionally, the use of glucose or galactose as the sole carbohydrate source does not reflect typical dietary patterns that include a variety of sugars, which further limits the generalisability of the findings to real-world diets.

Henning et al. carried out an open-label dietary intervention pilot study in 10 healthy lactating women (Table S1) [109]. Participants consumed 8 ounces of pomegranate juice daily for two weeks, following a two-week polyphenol-restricted washout period. HM samples were collected at baseline 3.7 ± 1.4 months postpartum and post-intervention. 16S rRNA gene sequencing was used to analyse bacterial profiles. The intervention resulted in an increased *Firmicutes*/*Faecalibacterium* ratio and reductions in the abundance of *Lactococcus* spp., *Subdoligranulum* spp., and *Acinetobacter* spp. These preliminary findings suggest that polyphenol-rich dietary components may modulate specific HM bacterial taxa, although the absence of dietary assessment limits interpretability. Notably, no dietary intake data were collected during either the washout or intervention period, making it difficult to confirm adherence to the polyphenol restriction or to account for other dietary factors that may have influenced the HM microbiota. In addition, the study lacked a control group, preventing comparisons with participants who did not receive the intervention and limiting the ability to attribute observed microbial changes specifically to pomegranate juice consumption.

Sindi et al. conducted a controlled longitudinal pilot dietary intervention in Australia involving 11 healthy lactating women [111]. At baseline 3.3 months postpartum, participants’ habitual dietary intake was assessed using three 24-h dietary recalls. Mothers were provided with pre-prepared nutritionally balanced (healthy) meals low in fat and sugar and high in fibre for a two-week intervention. HM samples were collected at four time points: baseline, immediately after the intervention, and 4 and 8 weeks post-intervention. Full-length 16S rRNA gene sequencing was used to profile the HM microbiota. The intervention resulted in small changes to the bacterial taxa in HM. An increase in *Cutibacterium acnes* and a decrease in *Haemophilus parainfluenzae* were observed post-intervention. By 4 weeks post-intervention, additional changes included increased abundance of *S. salivarius* and *S. parasanguinis*, along with elevated bacterial richness. At 8 weeks post-intervention, *H. parainfluenzae* and *S. parasanguinis* levels were significantly reduced while, *S. salivarius* abundance was increased. These findings suggest that short-term dietary modifications can influence the HM microbiota composition, with potential effects that may persist for weeks beyond the intervention period. While the use of pre-prepared meals during the intervention improved dietary control, the absence of a control group limits the ability to distinguish diet-induced effects from natural temporal variation in the HM microbiota, particularly given that maternal weight and BMI commonly decline during the first few months following delivery, which may influence the HM bacterial composition. Collectively, despite methodological limitations and contradictions reported in previous studies, most evidence supports the existence of an association between maternal diet and the bacterial composition of the HM.

## 6. Limitations of Current Studies

Despite offering valuable insights, both observational and interventional studies investigating maternal diet and the HM microbiota are limited by recurring methodological weaknesses such as small sample sizes, inappropriate dietary assessment, and variable milk collection protocols, which collectively reduce confidence in the consistency and generalisability of findings. These limitations are summarised in (Tables S1 and S2), which collectively function as a qualitative assessment, highlighting the key methodological weaknesses of the included studies. The quality of studies varied considerably, with small sample sizes, limited use of contamination controls, and inconsistent dietary assessment methods representing the most common issues. Together, these tables provide a transparent appraisal across critical domains of study design.

### 6.1. Small Sample Sizes and Underpowered Study Designs

Most of the current studies were small-scale pilot investigations involving 10–25 participants. This includes all interventional studies [109,111,114,116], as well as most observational ones (Tables S1 and S2) [50,51,73,106,107,108,110,112,113,115]. Small sample sizes reduce statistical power and limit the generalisability of findings across diverse populations. Additionally, 11 studies were observational [40,50,51,73,106,107,108,110,112,113,115], with nine of them employing cross-sectional designs [40,50,73,106,108,110,112,113,115], which limits the ability to establish causal relationships between maternal diet and the HM microbiota. In contrast, all interventional studies had short dietary interventions ranging from 30 h [116] to 88 ± 31 days [114], which may not have been sufficient to detect some of the microbial shifts in the HM composition. Exposure to longer-term dietary interventions may result in different outcomes thereby limiting the generalisability of these short-term findings. Moreover, none of the interventional studies incorporated a placebo control group, and two of these studies lacked any control group at all [109,111], further limiting the ability to attribute observed microbiota changes to the intervention itself rather than natural temporal postpartum variation. Taken together, the predominance of small pilot studies and short intervention durations means that current evidence is underpowered, leaving considerable uncertainty about the consistency and generalisability of associations between maternal diet and the HM microbiota.

### 6.2. Inconsistent Milk Collection Protocols

The protocol used for milk collection, including the application of aseptic technique, method of expression, and use of sterile equipment can significantly influence the composition of the HM microbiota [121]. Proper aseptic techniques, such as cleaning the breast, areola, and nipple with agents such as alcohol, chlorhexidine, or iodine followed by rinsing with sterile water or saline, aim to reduce contamination from maternal skin microbes. Additional practices include using sterilised or single-use pumps, wearing gloves or ensuring hand hygiene during hand expression, and discarding the initial few drops of milk to further minimise the influence of skin or environmental contaminants. Failure to implement or report these steps may compromise the reliability of microbiome analyses and lead to misinterpretation of contaminants as true members of the HM microbiota. However, not all of the studies described above provided clear or consistent details regarding their milk collection protocols. Five studies failed to mention whether nipple cleaning was performed [51,107,109,114,116], while others differed in the disinfectant used (Table S2). For example, Sindi et al. used a combination of 70% isopropyl alcohol and 2% chlorhexidine digluconate [111], while another study used an iodine swab [113]. Similarly, the method of milk expression (hand vs. pump) and sterilisation of pumps were inconsistently reported. Five studies reported using hand expression [106,110,111,112,113], whereas four employed sterilised breast pumps (Table S2) [50,51,73,116]. Three studies used both hand expression and breast pumps [40,108,115]. One of these studies reported that the pumps were heat-sterilised using microwave sterilisation bags [115], whereas the other two did not clarify whether the pumps were sterilised or if a single-use kit was used [40,108]. Additionally, three studies did not specify the method of expression at all, limiting the ability to assess the potential risk of contamination introduced during milk collection [107,109,114]. Overall, inconsistent or insufficiently reported milk collection protocols introduce uncertainty about whether observed microbial differences truly reflect the HM microbiota or are partly shaped by contamination, making cross-study comparisons difficult to interpret.

### 6.3. Limitations in Dietary Assessment Methods

Another recurring limitation is the failure to clearly report the timing of maternal dietary assessment in relation to HM sample collection. Without this temporal information, it becomes difficult to accurately interpret associations between maternal diet and the HM microbiota, particularly in cross-sectional studies. Three studies did not specify whether dietary intake data were collected concurrently with milk sampling, reducing confidence in linking dietary factors to the HM bacterial profiles (Table S1) [40,108,110]. In addition, one study did not assess maternal diet during the intervention or washout period limiting interpretation of the findings [109]. In the observational studies, dietary intake was primarily assessed using self-reported tools such as FFQs and 24-h dietary recalls, both of which are susceptible to recall bias [40,50,51,73,106,107,108,110,112,113,115]. Two studies used non-validated FFQs [73,106], while three studies used FFQs that were validated in populations that do not reflect the unique nutritional needs and intake patterns of pregnant or lactating women (Table S1) [40,110,113], reducing the reliability of their use in this context. Collectively, reliance on self-reported tools, variable timing of dietary assessment, and lack of validation in populations of pregnant or lactating women limit confidence in the accuracy of reported associations between maternal diet and the HM microbiota.

### 6.4. Limitations in Human Milk Microbiota Analysis

Several methodological inconsistencies across studies may limit the comparability and reliability of reported HM microbiota composition. One source of variation in HM microbiota profiles can be the milk fraction analysed. Across the reviewed studies, five removed the fat layer and analysed the skim fraction [40,50,51,111,115], three analysed the cell pellet [73,106,113], and two analysed the supernatant fraction [108,116], whereas five did not report which fraction was analysed (Table S2) [107,109,110,112,114], further contributing to methodological variability. Bacterial DNA composition has been reported to vary between skim and whole HM, though these differences are generally small [122]. Another study compared bacterial DNA profiles from the fat fraction, cell pellet, and whole milk and showed minor shifts in the relative abundances of detected taxa [123]. Similarly, when milk samples were separated into cell pellet and fat fraction and analysed separately, the bacterial DNA composition of the fat fraction differed slightly from that of the cell pellet [124]. These subtle differences, while small, highlight the importance of standardising milk fraction selection and clearly reporting the fraction analysed to minimise methodological variability and enable more robust cross-study comparisons.

Sequencing methodology also varied across studies. All 15 studies used 16S rRNA gene amplicon sequencing, but different hypervariable regions were targeted (V1–V2, V1–V3, V3–V4, and V4), which can lead to differing taxonomic resolution and bias in microbial community structure (Table S1) [125,126]. Only one study used full-length 16S rRNA sequencing [111], offering better taxonomic resolution. Additionally, three studies used short-amplicon 16S rRNA sequencing to infer microbial function [50,73,108]. This method is limited in that it detects only the 16S rRNA gene, but does not directly detect microbial genetic material and infers the functional profiles from taxonomic composition [127]. This functional inference is therefore less accurate than direct functional characterisation from metagenomic sequencing. Seferovic et al. was the only study to employ shotgun metagenomic sequencing, which allows direct profiling of both taxonomic and functional potential, providing more valid functional insights. Notably, they reported that maternal diet altered the functional potential of the HM microbiota [116]. These differences in sequencing approaches highlight the need for more comprehensive methodologies, such as shotgun metagenomics, to obtain accurate and functionally relevant insights into the impact of maternal diet on the HM microbiota. Moreover, contamination control is particularly important in the HM microbiota studies due to the low microbial biomass of HM [128]. However, contamination handling was inconsistent across the reviewed studies. Nine studies incorporated negative extraction and PCR controls [40,50,51,73,106,108,111,115,116], four included controls without reporting the results [50,51,73,113], and five failed to use contamination controls altogether (Table S1) [107,109,110,112,114]. In silico decontamination methods such as the decontam R package(v.1.12, v0.99.1, v. 1.1.0) were used by five studies [40,73,112,113,116], yet these post hoc approaches have limitations and may not fully account for reagent-derived contaminants [129]. Given the potential for contamination to obscure true microbial signals, the absence or insufficient reporting of negative controls raises concerns about the validity of reported findings. Taken together, the lack of standardisation in milk fraction analysed, sequencing methodologies, and inconsistent contamination control practices across the literature presents a substantial challenge to comparing HM microbiota studies, reduces the reliability of findings, and complicates interpretation of whether maternal diet is truly associated with the HM microbiota.

### 6.5. Variability in HM Lactation Stage and Lack of Standardisation

Milk samples were collected at various postpartum stages across studies. Colostrum is produced in the first few days postpartum and is rich in immunological components [130]. Transitional milk follows during days 5 to 14, gradually shifting in composition and mature milk is established thereafter [130]. Since microbial composition of the HM changes across lactation stages [33,34,42,43,44,45,46], observed diet-related differences in the HM microbiota may be confounded by these temporal shifts. Babakobi et al. and Bisanz et al. collected both transitional and mature milk [107,114], whereas Williams et al. collected milk samples spanning all three stages colostrum, transitional, and mature milk thereby introducing greater variability (Table S2) [51]. Another study failed to report the exact timing of milk sample collection, making it unclear whether the samples represented colostrum, transitional, or mature milk. [110]. Without standardising lactation stage at the time of sampling, it becomes difficult to determine whether observed microbial differences are due to maternal diet or natural changes in the HM microbiota composition over time. Therefore, because some studies collected samples across widely different lactation stages or did not report the timing of milk collection, it remains unclear whether observed microbial differences reported by reviewed studies are truly diet-related or instead reflect temporal shifts in the HM microbiota composition.

### 6.6. Inadequate Control for Confounding Variables

Six studies did not fully control for important confounders that are known to influence the HM microbiota such as antibiotic exposure [40,50,107,108,114,115], eight did not control for mode of delivery [50,51,73,108,111,113,114,115], and six did not control for infant feeding practices (Table S2) [51,73,106,108,112,115]. The extent to which confounding factors were addressed varied substantially across the reviewed studies, potentially affecting the validity of some observed associations between maternal diet and the HM microbiota. Eight studies used exclusion criteria to minimise potential confounding from maternal antibiotic use [51,73,106,109,110,111,112,113], while four studies excluded participants based on caesarean delivery [106,107,109,112]. However, exclusion criteria alone may not sufficiently control for confounding, especially for variables such as BMI, parity, or feeding practices, which require statistical adjustment [131]. Only three studies used multivariable models to control for such factors [40,50,110]. For example, LeMay-Nedjelski et al. and Moossavi et al. adjusted for maternal BMI and breastfeeding exclusivity [40,50] while, Londoño-Sierra et al. used maternal and infant variables as fixed factors and others as covariates in their analyses to reduce residual confounding (Table S2) [110]. Notably, Seferovic et al. accounted for genetic variation in secretor status by excluding non-secretors from metagenomic analysis (Table S2) [116]. In contrast, other studies, such as Shenker et al. and Williams et al., provided limited or no information on how confounding factors were managed [51,115]. Overall, inconsistent reporting and variable strategies for confounder control limit the ability to distinguish the effects of maternal diet from those of known confounders such as antibiotic exposure, delivery mode, and infant feeding practices, underscoring the need for future studies to standardise exclusion criteria and apply rigorous statistical adjustment methods to strengthen causal inference.

## 7. Biological Mechanisms and Clinical Implications

Two biological pathways may explain how maternal diet could shape the HM microbiome. One possibility is that maternal diet alters the maternal gut microbiota, which then changes the HM microbiota. While observational studies suggest that maternal diet may be linked to HM microbiota composition, such associations are limited by confounding and methodological variability [50,51,73,106,107,108,110,112,113]. Interventional studies, which offer stronger evidence of causality, are few in number and currently provide limited and inconclusive evidence [40,114,115,116]. Henning et al. reported significant compositional shifts in the HM microbiota following pomegranate juice supplementation. Although changes were observed in taxa such as (*Firmicutes*/*Faecalibacterium*, *Lactococcus*, *Subdoligranulum*, *Acinetobacter*), these are not core infant gut inhabitants [109]. In maternal stool, Henning et al. also reported modest increases in *Anaerostipes* and Sutterella, suggesting some responsiveness of the maternal gut microbiota to dietary intervention, yet the clinical significance of these changes remains uncertain [109]. Given that only a small number of HM-associated taxa, predominantly *Bifidobacterium* spp. have been reported to be able to colonise the infant gut [59,132], such findings are likely of limited clinical significance. In contrast, Sindi et al. observed small alterations in both maternal faecal and HM microbiota following a two-week intervention of reduced fat and sugar and increased fibre intake during lactation [111]. In the maternal gut, the relative abundance of *Bacteroides caccae* decreased and *Faecalibacillus intestinalis* increased, whereas in HM, *Haemophilus parainfluenzae* decreased and *Cutibacterium acnes* increased [111]. Again, the observed changes in the HM microbiota were confined to low-abundance taxa and those that are not typical residents of the infant gut reducing their clinical relevance. These shifts were not mirrored in the infant gut microbiota composition [133], which remained unchanged. However, despite the lack of compositional change, shotgun metagenomic analysis revealed a significant increase in the abundance of multiple bacterial metabolic pathways in the infant gut microbiome after the intervention, indicating functional modulation rather than taxonomic restructuring. This raises questions about the extent to which the entero-mammary pathway mediates dietary effects and highlights the importance of functional as well as compositional analyses. Similarly, Bisanz et al. found no impact of probiotic yogurt intake on either maternal gut or HM microbiota [114]. In line with these findings, Seferovic et al. demonstrated that maternal diet did not alter HM microbial taxonomy but provided a different perspective by showing that it influenced the functional potential of HM bacteria, in part through diet-driven modulation of HMO composition [116]. This underscores that maternal diet can change the HM functional capacity even without major taxonomic shifts, consistent with broader gut microbiome literature where function can change despite compositional stability [134,135]. These findings align with the observed stability of both maternal gut [136,137,138,139,140] and HM microbiota during lactation [38,51,67,117,137], suggesting resilience to short-term dietary perturbations. Functional redundancy within the maternal gut microbiota where different bacterial taxa perform overlapping metabolic functions may further buffer against compositional changes, leading to functional shifts rather than major taxonomic changes [141,142]. Taken together, findings from interventional studies suggest that maternal diet either does not significantly alter the maternal gut microbiota or alters it only slightly, leading to no or small-scale changes in the HM microbial composition.

Another possibility is that maternal diet can act directly by altering HM components such as HMOs, SCFAs, and AMPs [116,143]. These components can selectively favour or inhibit specific microbial taxa [144,145,146,147,148,149,150,151]. Many observational studies have shown associations between maternal diet and HMO profiles in HM [152,153,154]. However, only one interventional study showed that maternal diet can shape HMO profiles in HM [116]. Seferovic et al. provided evidence that diet can alter HMO profiles [116]. In their carbohydrate vs. fat cohort, a high-fat diet reduced total HMO-bound sialic acid compared to carbohydrate intake, whereas in the glucose vs. galactose cohort, glucose reduced total HMO-bound fucose relative to galactose. While individual HMOs did not consistently reach statistical significance, total fucosylation and sialylation patterns were significantly altered, underscoring that maternal macronutrient intake can shape HMOs composition. Similarly, another interventional study reported that maternal dietary intervention of reduced fat and sugar and increased fibre significantly altered the concentration of AMPs such as lactoferrin and lysozyme in HM [143]. Lactoferrin decreased by approximately 20% in week two compared to baseline, with daily variation also reduced across the intervention period. Lysozyme concentrations were reduced by 8–10% in week one but returned to baseline by week two, again with decreased daily variation [143]. However, no study has investigated the effect of maternal diet on SCFA profiles in HM. Dietary fibre intake increases SCFAs production in the gut [100,101,102,103,104,105], and these SCFAs, likely transferred Via maternal circulation [155,156,157], may rise in the HM following fibre-rich diets and subsequently influence the HM microbiota composition [158,159,160]. However, this pathway remains poorly characterised. Overall, these findings indicate that maternal diet may influence HM bioactive components such as HMOs, SCFAs, and AMPs more readily than microbial taxa, pointing to functional modulation as the most plausible pathway of diet–HM interactions, though this remains insufficiently explored and requires confirmation in larger, well-controlled interventional studies.

## 8. Future Directions

The methodological limitations discussed above underscore the need for larger, rigorously designed randomised controlled trials with longer intervention durations, robust dietary monitoring, and comprehensive microbiological quality control. Future research should aim to overcome current barriers to causal inference and reproducibility through methodological standardisation, expanded sample sizes, and advanced analytical approaches.

First, adequately powered multicentre and longitudinal trials are essential to capture the temporal dynamics of the HM microbiota and assess the sustained impact of maternal diet over different lactation stages. Recruiting culturally diverse cohorts will be critical to ensure that findings are generalisable across populations with varying diets and genetic backgrounds. While such studies are logistically complex and resource-intensive, strategic collaboration between research centres could help reduce costs and improve feasibility. Second, standardisation of milk collection protocols is important. This should include explicit guidelines for aseptic technique, method of expression, and equipment sterilisation, alongside clear reporting in publications. International consensus statements, similar to those developed for gut microbiome research, could be adapted for HM studies to facilitate harmonisation across research groups. Third, robust and accurate dietary assessment remains a priority. The incorporation of dietary biomarkers such as fatty acid profiles, isotope ratios, metabolomic signatures can complement and validate self-reported tools such as FFQs and 24-h recalls, thereby improving accuracy and reducing recall bias. However, cost and technical expertise requirements may limit widespread use. Pilot implementation in selected cohorts could demonstrate feasibility before broader implementation. Fourth, to advance understanding of functional as well as taxonomic profiles, future studies should utilise integrated multi-omics approaches combining metagenomics, metabolomics, lipidomics, and transcriptomics rather than relying on 16S rRNA sequencing only. Long amplicon sequencing should be used to better study the relationship between maternal diet and the HM microbiota. Functional profiling should preferably be performed using shotgun metagenomics rather than inferred from taxonomic data to more accurately characterise the microbial function. Fifth, future studies must prioritise comprehensive contamination control in HM microbiome research due to the low microbial biomass of milk. This includes the use of negative extraction and PCR controls, rigorous in silico decontamination, and transparent reporting of control results. Sixth, future dietary intervention trials should explicitly include microbiome endpoints and explore personalised lactation nutrition strategies informed by maternal metabolic status, microbiome composition, and infant health outcomes. These precision approaches, while promising, will require careful ethical consideration, especially in low-resource settings, to avoid exacerbating health disparities. Seventh, future studies should go beyond taxonomy profiling and incorporate detailed measurements of HM metabolites and bioactives such as HMOs, SCFAs, and AMPs to clarify how maternal diet influences both the composition and function of the HM microbiome, and ultimately infant health. Finally, addressing confounding factors in future work will require moving beyond exclusion criteria to robust statistical modelling, with adjustment for variables such as maternal BMI, parity, antibiotic exposure, mode of delivery, and infant feeding practices. Harmonising covariate selection across studies could further improve comparability and meta-analytic potential. Collectively, implementing these methodological improvements will be essential for generating robust, generalisable evidence to inform targeted dietary recommendations that optimise the HM microbiota for the purpose of improving infant health.

## 9. Conclusions

Current evidence indicates a potential link between maternal diet and the composition and functional potential of the HM microbiota, yet extensive methodological heterogeneity and limitations across existing studies hinder definitive conclusions and complicates synthesis of results. The predominance of small, underpowered studies, accompanied by inconsistent dietary assessment and microbiome analysis methods, limits the comparability and reproducibility of findings. Standardisation of milk collection protocols, rigorous contamination control, culturally inclusive recruitment, and the incorporation of objective dietary biomarkers will be critical in advancing this field. Future research should prioritise well-powered, multicentre randomised controlled trials with extended intervention durations, integrating multi-omics platforms to capture both taxonomic and functional shifts. Such approaches, alongside robust confounder control and harmonised reporting standards, will be essential to establish causal relationships. At present, findings should be viewed as preliminary, and more rigorous evidence is required before maternal diet can be translated into clinical nutrition strategies. Ultimately, generating high-quality evidence in this field offers a promising avenue to inform maternal nutrition strategies aimed at optimising the HM microbiota and, in turn, supporting healthy infant development.

## Data Availability

No new data were created or analyzed in this study.

## Supplementary Materials

The following supporting information can be downloaded at: https://www.mdpi.com/article/10.3390/microorganisms13102347/s1, Table S1: Summary of studies examining the association between maternal diet and the human milk microbiota; Table S2: Study characteristics, methodological considerations, and main conclusions for studies looking at the association between maternal diet and the human milk microbiota.
