# Supplementary material for: The Association Between Maternal Diet and the Human Milk Microbiome: A Review of Evidence and Methodological Challenges
Source: Microorganisms. 2025 Oct 14;13(10):2347. doi: 10.3390/microorganisms13102347 (PMC12566554; doi:10.3390/microorganisms13102347)
Supplement: Supplementary file 1 [file microorganisms-13-02347-s001.zip › microorganisms-3853320-supplementary.pdf]

Table S1: Summary of studies examining the association between maternal diet and the human milk microbiota.

Study reference	Participants, samplesize (n), infant gestational age at delivery, country	Study Design	Time of maternal diet assessment	Maternal diet assessment	Time of human milk sampling	Methods for human milk microbiome analysis	Maternal dietary factor	Outcome of human milk microbiota
Bzikowska-Jura et al., 2025	Healthy lactating women (15), full-term infants, Poland.	Cross-sectional (pilot study).	4-6 weeks postpartum.	Food frequency questionnaire to assess 3 months prior to milk sampling (last 2 months of pregnancy and one month of lactation) and 3-day dietary record (for current intake).	4-6 weeks postpartum.	16S rRNA gene sequencing (V3–V4 region)<br><br>**Contamination controls:** A negative extraction control and PCR-negative and positive controls were included and sequenced. The Decontam package was used to remove potential contaminants from the dataset**.**	↑Starch, vitamin A, beta-carotene, total carbohydrates, MUFA, animal protein	↑Firmicutes
							↑Retinol	↑Bacteroidota
							↑Phosphorus, potassium, total carbohydrates, MUFA, total fat	↑*Enterococcaceae*
							↑Lactose	↑*Streptococcaceae*
							↑Vitamin B12	↑*Sphingomonadaceae*
							↑Manganese	↑*Sphingobacteriaceae*
							↑Calcium, sucrose	↑*Moraxellaceae*
							↑PUFA	↓*Moraxellaceae*
							↑LC-PUFA	↓*Pseudomonadaceae*
							↑Animal milk	↑*Sphingomonadaceae*
							↑Plant oils	↑*Staphylococcaceae*
							↑Buckwheat, peanuts	↑*Sphingobacteriaceae*

							↑Peanuts	↑*Xanthomonadaceae*
							↑Wholegrain	↑*Sphingobacteriaceae,* ↑*Xanthomonadaceae,* ↑*Enterobacteriaceae*
							↑Oily fish, lean fish, butter	↑*Bacillaceae*
Ajeeb et al., 2024	Healthy lactating women (64), gestational age not reported, Guatemala	Cross-sectional	Day 6-46 postpartum and day 109-184 postpartum.	Two non-consecutive 24-h dietary recalls.	The same day as the second 24-h dietary recall at day 109-184 postpartum.	16S rRNA gene sequencing (V1–V3 region.<br><br>**Contamination controls:**<br>The Decontam package was used to remove potential contaminants from the dataset. Prevalence and sparsity filtering and ordination analysis were also applied.	↑Pantothenic acid, choline, saturated fat, cobalamin, riboflavin, cholesterol, vitamin D	↑*Streptococcus salivarius, Streptococcus*_MS_12, *Corynebacterium*_1, *Kocuria palustris, Brevundimonas*_MS_1.<br>Several other taxa also showed significant associations.
Sindi et al., 2024	Healthy lactating women (11), full-term infants, Australia	Controlled dietary intervention, longitudinal, pilot study.<br><br>Lactating mothers consumed a reduced fat and sugar and increased fibre diet for 2 weeks.	Pre-intervention (baseline): 3.3 months postpartum and during the 2-week intervention.	Pre-intervention: Three 24-h dietary recalls.<br><br>During-intervention: pre-prepared healthy meals of known nutrient levels were consumed.	Pre-intervention (baseline): 3.3 months postpartum, post-intervention, 4 weeks post-intervention, 8 weeks post-intervention.	Full-length 16S rRNA gene sequencing.<br><br>**Contamination controls:**<br>Negative extraction and PCR controls were included. ArcticZymes PCR decontamination kit to reduce contaminating DNA in PCR master mixes.	↓Fat, ↓saturated fat, ↓sugar; ↑fiber	Post-intervention: ↑*Cutibacterium acnes,* ↓*Haemophilus parainfluenzae*<br><br>4 weeks post-intervention: ↑*Cutibacterium acnes,* ↓*Haemophilus parainfluenzae,* ↑*Streptococcus salivarius, and* ↑*Streptococcus parasanguinis,* and ↑ bacterial richness.<br><br>8 weeks post-intervention:

								↓*Haemophilus parainfluenzae,* ↑*Streptococcus salivarius,* and ↓*Streptococcus parasanguinis.*
Londoño-Sierra et al., 2023	Healthy lactating women (30), full-term infants, Colombia	Cross-sectional.	Not reported.	Two non-consecutive 24-h dietary recalls to assess maternal diet during first trimester of lactation and food frequency questionnaire to assess maternal diet during the last two trimesters of pregnancy period.	First trimester of lactation.	16S rRNA gene sequencing (V3–V4 region).<br><br>**Contamination controls:** None.	↑Simple carbohydrates	↑*Enterobacter* ↓*Bifidobacterium*
							↑Total fat, ↑Saturated fat, ↑Monounsaturated fat	↑*Eubacterium* ↓*Bifidobacterium*
							↑Folic acid	↑*Akkermansia*
							↑B complex vitamins (B1, B2, B3)	↑*Gemella*
							↑Vitamin A	↑*Bifidobacterium* ↑*Corynebacterium* ↑*Ruminococcus*
							↑Total protein and total carbohydrates.	↑*Enterobacter*
							↑Total protein, total carbohydrates, cholesterol, dietary fiber, vitamin A, vitamin C, folic acid, pantothenic acid, magnesium	↓*Aerococcus*
							↑Saturated fat	↑*Enterobacter*, *Halomonas.* ↓*Corynebacterium*, *Cutibacterium*, *Escherichia-Shigella*, *Lachnospiraceae* NK4A136
							↑Dietary fiber	↓*Enterobacter*
							↑Zinc	↑*Pseudomonas*
							↑Vitamin C	↑Rothia
							↑Dietary fiber	↓Ruminococcaceae, *Ruminiclostridium,* Ruminococcus 1
							Total protein	↓*Bifidobacterium*

Henning et al., 2022	Healthy lactating women (10), full-term infants, USA	Open-label dietary intervention, pilot study.<br><br>Lactating mothers consumed 8 oz of pomegranate juice daily for 2 weeks following a 2-week low-polyphenol washout phase.	Not assessed; no dietary intake data collected.	Not assessed; no dietary intake data collected.	Pre-intervention (baseline): 3.7 ± 1.4 months postpartum.<br><br>Post-intervention: 15 days following the start of the dietary intervention.	16S rRNA gene sequencing (V4 region).<br><br>**Contamination controls:** None	Daily intake of 8 oz pomegranate juice for 2 weeks.	↑Firmicutes/*Faecalibacterium*<br>↓*Lactococcus,*<br>↓*Subdoligranulum,*<br>↓*Acinetobacter*
Marsh et al. 2022	Healthy lactating women (72), full-term infants; USA.	Cross-sectional.	Not reported.	Food frequency questionnaire.	≥2 weeks postpartum.	16S rRNA gene sequencing (V4 region).<br><br>**Contamination controls:** Negative extraction control was included.	Omnivore diet.	↑*Vermiphilaceae, Dietzia, Mycobacterium, Rothia, Prevotellaceae* NK3B31 group, *Symplocastrum* CPER-KK1, *Promicromonospora, Geobacillus, Acidobacteriaceae* Subgroup_1, *Actinomyces, Brevibacillus, Candidatus Stoquefichus, Prevotella, Dermabacter, Bilophila.*
							Vegan diet.	↑*Muribaculum, Halobacillus, Clostridium spiroforme, Cloacibacterium.*

Seferovic et al. 2020	Healthy lactating women (14), full term infants; USA.	Two single-blind, randomised cross-over dietary intervention study.<br><br>Glucose vs. galactose diet Glu/Gal cohort (n=7) for 30-57 hours.<br><br>high fat vs. high carbohydrate diet Carb/Fat cohort (n=7) for 8 days.	Glu/Gal cohort: 9.3±1.4 weeks postpartum.<br><br>Carb/Fat cohort: 10.1±0.9 weeks postpartum.	NA.	Glu/Gal Cohort: at the end of each 30-57 hour dietary intervention.<br><br>Carb/Fat Cohort: at the end of each 8-day dietary intervention, specifically days 5-8.	Shotgun metagenomic sequencing and 16S rRNA gene sequencing (V1–V3 region).<br><br>**Contamination controls:** Negative extraction control was included. The decontam package was used to remove potential contaminants.	Glu/Gal cohort: isocaloric and isonitrogenous drinks containing either glucose or galactose as the sole carbohydrate source.<br><br>Carb/Fat cohort: high-carbohydrate (60% CHO) or high-fat (55% fat) diet.	Diet significantly altered milk metagenomic (functional) profile but not taxonomic composition.
Shenker et al. 2020	Healthy lactating women (62), gestational age not reported, UK.	Cross-sectional.	3-48 months postpartum.	Food frequency questionnaire.	3-48 months postpartum.	16S rRNA gene sequencing (V1–V2).<br><br>**Contamination controls:** Negative extraction controls were included.	Intake of alcohol, fruit, vegetables, meat, fish, egg, dairy, soya, supplements (vitamin D, folate,and calcium).	No significant association of diet with the human milk taxonomic profiles.
Babakobi et al. 2020	Healthy lactating women (22), full term infants, Israel	Prospective, longitudinal, study.	3 months postpartum	Food frequency questionnaire at 3 months postpartum to assess maternal diet during the pregnancy period and the first 3 months of lactation	1 week, 1 month, and 3 months postpartum	16S rRNA gene sequencing (V3–V4 region)<br><br>**Contamination controls:** None	↑Total polyunsaturated fat	↓*Streptococcus*
							↑Total monounsaturated fat	↓*Streptococcus*
							↑Folic acid	↓*Streptococcus*
							↑Vitamin B12	↑ *Streptococcus*
Cortes-Macías et al. 2020	Healthy lactating women (120), full term infants, Spain	Cross-sectional.	Day 11 (±4) postpartum	Food frequency questionnaire	Day 11 (±4) postpartum	16S rRNA gene sequencing (V3–V4 region)<br><br>**Contamination controls:**<br>Negative extraction and PCR controls were	↑Carbohydrates	↑*Staphylococcus*<br>↓*Klebsiella* and *Enterobacter*
							↓Total protein	↑*Staphylococcus*
							↑Total protein, EPA, DPA, selenium, and zinc	↑*Streptococcus*

						included. ASVs with a relative abundance of less than 0.01% and those present less than 3 times in ≥20% of samples were removed. The decontam package was used to remove potential contaminants from the data set.		
							↑Carbohydrates and polyphenols	↑*Bifidobacterium*
							↓Total lipid	↑*Bifidobacterium*
							↑Total animal protein and saturated fat	↓*Enterococcus*
							↑Total animal protein, DHA, EPA, and DPA	↑*Gemella*
							↑Total lipid	↑*Klebsiella* and *Enterobacter*
							↑Calcium	↓*Veillonella and* ↑*Stenotrophomonas*
							↑Total fibre, plant protein, and insoluble dietary fibre	↑*Veillonella*
							↑Vitamin A	↑*Enterococcus*
							↓Vitamin D	↑*Enterococcus*
LeMay-Nedjelski et al. 2020	Normoglycemic lactating women (56), lactating women with gestational diabetes mellitus (21), or lactating women with impaired glucose tolerance (16), full term infants, Canada	Cross-sectional.	3 months postpartum	Food frequency questionnaire	3 months postpartum	16S rRNA gene sequencing (V4 region)<br><br>**Contamination controls:** A negative extraction control was included. OTUs present in less than 3 samples were removed.	↑Fibre from grains	↓Fusobacteria and ↑*Acinetobacter*
							↑Total fibre	↓*Streptococcus*
							↑Trans fats	↑*Staphylococcus* and *Gemella*
							↑Monounsaturated fat	↑*Acinetobacter* and *Gemella*
							↑Polyunsaturated fat	↓*Acinetobacter*
Padilha et al. 2019	Healthy lactating women (94), full term infants, Brazil	Cross-sectional.	Day 7 (±3) and 30 (±4) postpartum	24-h dietary recall at day 7 (±3) and day 30 (±4) postpartum to assess maternal diet during the lactation period	Day 30 (±4) postpartum	16S rRNA gene sequencing (V4 region)<br><br>**Contamination controls:**	↑Vitamin C during pregnancy	↑Cluster driven by *Staphylococcus*
							↑ Polyunsaturated fat and linoleic fatty acid during lactation	↑*Bifidobacterium*

				Food frequency questionnaire at day 30 (±4) postpartum to assess maternal diet during the pregnancy period		Negative extraction and PCR controls were included. OTUs present in less than 3 samples and those with a relative abundance higher than 0.5% across all samples were removed.	↓Sugars during lactation	↑*Pseudomonas*
							↑Vitamin B9 during lactation	↑*Pseudomonas*
							↑B vitamins particularly (B1, B2, and B9) during lactation	↓*Enterococcus*
Moossavi et al. 2019	Healthy lactating women (393), term infants, Canada	Cross-sectional.	Not reported.	Food frequency questionnaire.	3-4 months postpartum.	16S rRNA gene sequencing (V4 region).<br><br>**Contamination controls:**<br>Negative and positive PCR controls were included. The decontam package was used to remove potential contaminants from the data set. ASVs with less than 20 reads across dataset were removed.	Not reported.	Maternal diet was not directly associated with the human milk microbiota.
Williams et al. 2017	Healthy lactating women (21), gestational age not reported, USA	Prospective longitudinal study.	Days 2, 5, and 10 (±1 d) and 1, 2, 3, 4, 5, and 6 months (±1 d) postpartum	24-h dietary recall	Days 2 and 5 (colostrum), and 10 (±1 d) and 1, 2, 3, 4, 5, and 6 months (±1 day) postpartum	16S rRNA gene sequencing (V1–V3 region)<br><br>**Contamination controls:**<br>A negative extraction control was included.	↑Saturated and monounsaturated Fat	↓*Corynebacterium*
							↑Unsaturated and Polyunsaturated fat	↑*Proteobacteria*
							↑Total carbohydrates	↓Firmicutes, ↑Bacteroidetes, and *Gemella*
							↑Insoluble fibre	↑*Rothia*
							↑Total protein intake	↑*Gemella*
							↑Essential amino acids	↑Proteobacteria
							↑Pantothenic acid	↓*Streptococcus*
							↑Riboflavin and calcium	↑*Veillonella*
							↑Thiamin, niacin, folate, vitamin B-6, and chromium	↓*Lactobacillus*

Bisanz et al. 2015	Healthy lactating women (15), term infants, Tanzania	Open-label, dietary interventional, pilot study.<br><br>6 Participants consumed 250 g of probiotic yogurt daily, 6 days per week, during the last two trimesters of pregnancy and for 1 month postpartum.<br><br>Control group included 9 participants (no intervention).	Gestational week 21 ± 4, 32 ± 2, and 1 week to 1 month postpartum.	48-h dietary recall.	1 week to 1 month postpartum.	16S rRNA gene sequencing (V4 region).<br><br>**Contamination controls:** OTUs were filtered to retain taxa composing ≥0.01% of total reads. Taxa with zero counts were removed**.**	Moringa-supplemented probiotic yogurt that contained approximately 10^{10} CFU of *Lactobacillus rhamnosus* GR-1 per 250 g serving. The yogurt provided approximately 9.95 g of protein, 385 mg of calcium, 269 µg of vitamin A (retinol activity equivalents), 1.1 mg of vitamin B2, and 1.2 mg of iron.	No significant differences in human milk microbiota diversity or composition between intervention and control groups.

MUFA: monounsaturated fatty acids, PUFA: polyunsaturated fatty acids, LC-PUFA: long chain polyunsaturated fatty acids.

Table S2: Study characteristics, methodological considerations, and main conclusions for studies looking at the association between maternal diet and the human milk microbiota.

Study reference	Lactation stage at sampling	Infant feeding type	Milk collection protocol	Milk processing protocol	Milk fraction analysed	Confounding factors controlled for	Potential sources of bias (limitations)	Main author conclusion
Bzikowska-Jura et al., 2025	Mature milk.	Exclusive breastfeeding.	Hand expression, breasts cleaned with iodine swab.	**Storage:** immediately frozen at −20 °C, then stored at −80 °C.<br><br>**DNA extraction volume:** Not reported.	Pellet.	Excluded: Maternal antibiotics/probiotics in past 3 months, parity, smoking, chronic diseases.	Small sample size, observational design, combined dietary assessments pregnancy and lactation periods, results of negative controls not reported in microbiota analysis.	Maternal diet is associated with changes in the composition of HM microbiota. Specific nutrient and food group intake correlated with key bacterial taxa.
Ajeeb et al., 2024	Mature milk.	Exclusive or predominant breastfeeding (includes use of traditional "agüitas" for 6 months).	Hand-expression, nipple cleaned with 70% ethanol.	**Storage:** stored at −30°C in the field laboratory, then shipped and stored at −80 °C.<br><br>**DNA extraction volume:** 1 mL used.	Not reported.	Excluded: 9aesarean deliveries, maternal antibiotics, subclinical mastitis, non-singleton birth.	Small sample size; observational design, no control for breastfeeding exclusivity, HM fraction not reported, no contamination controls.	Maternal diet is associated with changes in the composition of HM microbiota.
Sindi et al., 2024	Mature milk.	Exclusive breastfeeding.	Hand expression, nipple and areola cleaned with 70% isopropyl alcohol + 2% chlorhexidine digluconate.	**Storage:** home freezer ≤7 days, then −80 °C.<br><br>**DNA extraction volume:** 2 mL used.	Skim fraction. Fat layer was removed.	Excluded: maternal disease, antibiotics, pregnancy complications, multiple pregnancies, dietary restrictions, early solid introduction.	Small sample size, no control group.	A 2-week low-fat, low-sugar, high-fiber intervention during lactation is associated with small but significant changes in the HM microbiota, including increased diversity and specific taxonomic shifts; some changes persisted up to 8 weeks post-intervention.
Londoño-Sierra et al., 2023	Could be colostrum, transitional, or mature milk (authors did not specify the exact time of milk sampling).	Exclusive breastfeeding.	Hand expression, nipple cleaned with 0.5% chlorhexidine.	**Storage:** stored at −80 °C.<br><br>**DNA extraction volume:** 6-10 mL used.	Not reported.	Excluded: Antibiotics, antidepressants, laxatives, corticosteroids, proton pump inhibitors, probiotics, smoking, alcohol, obesity (BMI	Small sample size, observational design, timing of maternal diet assessment and HM sampling not reported, HM fraction not reported, no contamination controls.	Maternal diet during gestation and lactation are associated with the HM microbiota composition.

						> 30), underweight (BMI < 18.5).<br><br>Controlled: Statistical adjustment for multiple maternal and infant variables by entering each variable as a fixed factor and others as covariates to reduce confounding effects.		
Henning et al., 2022	Mature milk.	Exclusive breastfeeding.	Not reported.	**Storage:** not reported.<br><br>**DNA extraction volume:** 5 mL used.	Not reported.	Excluded: cesarean deliveries, antibiotics, laxatives, pre/probiotics, anti-inflammatory drugs, allergies to pomegranate juice.	Small sample size, no control group, no dietary data collected during washout or intervention, expression method, nipple cleaning, and HM fraction not reported, no contamination controls.	A 2-week pomegranate juice intervention during lactating was associated with changes in the HM microbiota.
Marsh et al. 2022	Mature milk.	Not reported.	Hand and pump expression, nipple cleaning not reported.	**Storage:** stored at -20 °C.<br><br>**DNA extraction volume:** 2 mL used.	Supernatant.	Excluded: pregnancy, various chronic conditions.	Small sample size, observational design, time of dietary assessment not reported, varied expression methods (hand and breast pump), exclusive breastfeeding status not specified, no control for antibiotic exposure, mode of delivery, or infant feeding practices.	Vegan, vegetarian, and omnivore maternal diets are associated with the HM microbiota.
Seferovic et al. 2020	Mature milk.	Exclusive breastfeeding.	Pump expression, pumps were sterilised by autoclaving, nipple cleaning not reported.	**Storage:** stored at −80 °C, 2-3 freeze–thaw cycles.<br><br>**DNA extraction volume:** 1 mL used.	Supernatant.	Excluded: non-secretors excluded from metagenomic analysis.<br><br>Cross-over design inherently controlled for maternal factors such as BMI, geography, mode of delivery, and antibiotics.	Small sample size, no placebo control group, inability to assess gene expression or protein activity.	Dietary intervention significantly altered HM metagenomic (functional) profile but not taxonomic composition.

Shenker et al. 2020	Mature milk.	Not reported.	Hand and pump expression, pumps were sterilised using microwave bags, breasts not cleaned.	**Storage:** stored at −80 °C within 30 min.<br><br>**DNA extraction volume:** 2 mL used.	Skim fraction. Fat layer was removed.	Not reported.	Small sample size, observational design, broad and variable timing of dietary assessment and HM sample collection (3-48 months postpartum), exclusive breastfeeding status not specified, no control for antibiotic exposure, mode of delivery, or infant feeding practices.	No significant association of diet with the HM taxonomic profiles.
Babakobi et al. 2020	Transitional and mature milk.	Exclusive breastfeeding.	Not reported.	**Storage:** stored at −20 °C.<br><br>**DNA extraction volume:** 400 µL used.	Not reported.	Excluded: Any diseases, cesarean deliveries, medication use.	Small sample size, observational design, combined dietary assessments pregnancy and lactation periods, HM fraction and nipple cleaning not reported, no contamination controls, no control for antibiotic exposure and lactation stage.	Maternal dietary patterns are associated with the human milk specific microbial profiles.
Cortes-Macías et al. 2020	Transitional milk.	Not reported.	Sterile pump expression, breast cleaned with soap or 0.5% chlorhexidine.	**Storage:** stored at −80 °C.<br><br>**DNA extraction volume:** 1.5-2 mL used.	Pellet.	Excluded: any disease, medication use, pro/prebiotics, with the exception of antibiotic exposure during pregnancy and/or at delivery.	Small sample size, observational design, not validated FFQ, results of negative controls not reported in microbiota analysis. no control for mode of delivery or infant feeding practices.	Maternal diet shapes the HM microbiota composition.
LeMay-Nedjelski et al. 2020	Mature milk.	Exclusive breastfeeding (50.5%) and breastfeeding + formula (49.5%).	Sterile pump expression, breasts not cleaned.	**Storage**: stored at −80 °C.<br><br>**DNA extraction volume:** 1 mL used.	Skim fraction. Fat layer was removed.	Excluded: diabetes and use of insulin.<br><br>Controlled: statistical adjustment for confounders such as pre-pregnancy BMI, complete breast expression, DNA extraction batch, PCR sequencing batch, breastfeeding exclusivity, direct breastfeeds per day.	Small sample size, observational design, milk not aseptically collected, results of negative controls not reported in microbiota analysis, no subgroup analyses for women with different glucose intolerance conditions, no control for antibiotic exposure, mode of delivery.	Maternal dietary intake of fat and fiber is associated with HM microbiota composition.

Moossavi et al. 2019	Mature milk.	Exclusive breastfeeding (48.3%) and partial breastfeeding supplemented with infant formula or solid food (51.7%).	Hand and pump expression.	**Storage:** refrigerated ≤24 h, then stored at −80 °C.<br><br>**DNA extraction volume:** 1 mL used.	Skim fraction. Fat layer was removed (pellet used for DNA extraction).	Controlled: statistical adjustment for confounders such as infant sex, mode of delivery, mode of feeding, parity.	Cross-sectional design, timing of dietary assessment not reported, milk samples were not aseptically collected, method of milk expression varied.	Maternal diet was not directly associated with HM microbiota composition.
Padilha et al. 2019	Mature milk.	Exclusive breastfeeding (83%) and breastfeeding + formula (17%).	Hand expression, nipple cleaned with 1% chlorhexidine.	**Storage:** on ice ≤4 h, then stored at −80 °C.<br><br>**DNA extraction volume:** 1.5 mL used.	Pellet.	Excluded: cesarean deliveries, antibiotics, pre/probiotics, medication use, laxatives, chronic disease.	Small sample size, observational design, not validated FFQ, no control for infant feeding practices.	Maternal diet during pregnancy and lactation is associated with the HM microbiota composition.
Williams et al. 2017	Colostrum, transitional, and mature milk**.**	Not reported.	Pump expression, sterile single-use kit, nipple cleaning not reported.	**Storage:** on ice ≤1 h, then stored at −80 °C.<br><br>**DNA extraction volume:** 0.5-10 mL used.	Skim fraction. Fat layer was removed.	Excluded: Antibiotics.	Small sample size, observational study, nipple cleaning not reported, results of negative controls not reported in microbiota analysis, values for both dietary intake and microbial profiles were averaged across all time points, no control for lactation stage.	HM microbiota composition is relatively stable over lactation, but it is associated with maternal diet.
Bisanz et al. 2015	Transitional and mature milk.	Not reported, assumed all exclusively breastfed.	Not reported.	**Storage:** stored at −80 °C.<br><br>**DNA extraction volume:** 500 μL used.	Not reported.	Excluded: HIV positive samples were excluded.	Small sample size, no placebo control group, variability in intervention duration (88 ± 31 days), variability in timing of HM sample collection (1 week to 1 month postpartum), no control for lactation stage, antibiotic exposure, or mode of delivery, nipple cleaning not reported, method of expression not reported, HM fraction not reported, no contamination controls.	Moringa-supplemented probiotic yogurt does not significantly alter the HM microbiota.

HM: human milk; FFQ: food frequency questionnaire.
